# The Diagnostic Utility of Early Ct Brain Imaging in Poisoned Intensive Care Patients

**DOI:** 10.1186/2197-425X-3-S1-A498

**Published:** 2015-10-01

**Authors:** M Henderson, R Docking, M Hughes

**Affiliations:** Glasgow Royal Infirmary, Intensive Care Unit, Glasgow, United Kingdom; Western Infirmary, Intensive Care Unit, Glasgow, United Kingdom

## Introduction

Computed tomography (CT) brain imaging is a valuable resource for investigating unconscious patients. Despite the potential benefits of identifying coincident pathology and sequelae, early CT scans are rarely positive in comatose poisoned patients and their routine use is controversial[[1]].

## Objectives

To determine the number of patients admitted to the Intensive Care Unit (ICU) at Glasgow Royal Infirmary after poisoning, identify those who received CT brain imaging and assess its utility in each case.

## Methods

Cases were identified retrospectively using the WardWatcher™ database. Patients were included if they were admitted to ICU after deliberate, accidental or recreational poisoning. Those with toxicity from routine drug therapies were excluded. Approval was gained from the Caldicott Guardian.

## Results

Between January 2011 and February 2014, 110 patients were admitted to ICU after poisoning (3.7% of admissions). the median age was 38 years (range 15-67) with a median APACHE II score of 18 (range 3-41). 48 patients (43.6%) had an early CT brain scan. the result was unremarkable for acute pathology in 44 patients (91.7%) including 3 benign incidental findings. 4 early scans were abnormal with 3 significant incidental findings and 1 relevant abnormal result (cerebral oedema). 3 patients (2.7%) required a scan later in their ICU stay - only one was abnormal, confirming a clinical deterioration. 4 patients died (3.6%) in hospital, all while in ICU, and each patient had received an early CT scan.

The most common indication for early CT scanning was reduced consciousness. the median Glasgow Coma Score (GCS) in the scanned cohort was 6/15.

Co-ingestion of multiple drugs including alcohol was common - 86 patients (78.1%) had a mixed overdose.

## Conclusions

In our study, early CT brain imaging had a very low diagnostic yield, with only one early scan (2.1%) revealing a relevant finding. the incidence of late scanning was low and all patients who died received early CT scans. This suggests that no acute pathology was missed. This corresponds with previous research. a Scottish study found that only 3.6% of CT scans were abnormal [[2]]. We believe that early CT scans should not be routine in comatose patients with an otherwise uncomplicated overdose. However, it is prudent to scan patients for specific associated indications such trauma, abnormal neurological signs, seizures or an uncertain history of drug ingestion. Existing guidelines triage patients to receive CT scans in other clinical scenarios. the evolution of similar guidelines in poisoning would help to ensure standardised care and the efficient use of resources.

## Grant Acknowledgment

No funding was sought.

**Figure 1:**
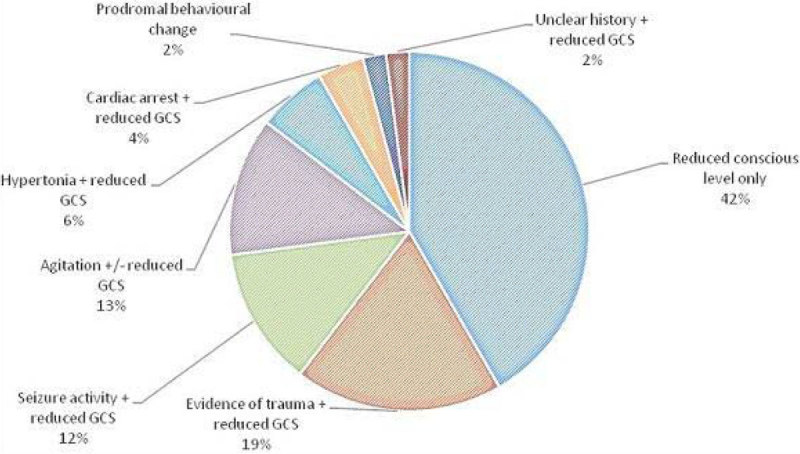
***Indications for early CT scanning.***

**Table 1:** *Results of early CT scanning.*

CT result	Study Classification	Number
Normal	Unremarkable	41
Cerebral atrophy	Unremarkable	2
Pineal cyst - known, unchanged	Unremarkable	1
Cerebral oedema	Abnormal	1
Previous cortical infarct	Abnormal	1
Mega cisterna magna	Abnormal	1
Tonsillar descent - Arnold Chiari Type 1 Malformation or Tonsillar Ectopy suspected	Abnormal	1

**Table 2:** *Most common poisons by class.*

Pharmacological class	Number
Antidepressants	60
Ethanol	49
Benzodiazepines	23
Opioids (excluding heroin)	21
Illicit drugs (including heroin)	17
Paracetamol (acetaminophen)	15
Atypical antipsychotic	12
Antiepileptic	12
Other sedative	7
